# Smart Energy Management in Agricultural Wireless Sensor Nodes Using TinyML-Based Adaptive Sampling

**DOI:** 10.3390/s26072014

**Published:** 2026-03-24

**Authors:** Adrian Hinostroza, Jimmy Tarrillo, Moises Nuñez

**Affiliations:** Department of Electrical and Mechatronics Engineering, Universidad de Ingeniería y Tecnologia—UTEC, Lima 15063, Peru; jtarrillo@utec.edu.pe (J.T.); mnunez@utec.edu.pe (M.N.)

**Keywords:** TinyML, adaptive sampling, wireless sensor network, energy efficiency, smart agriculture

## Abstract

Smart sensors are increasingly used in agriculture to monitor environmental conditions and support data-driven decision-making. However, traditional sensor implementations face critical challenges related to power consumption, especially in remote farms—such as pitaya plantations—where access to electricity and ongoing maintenance is limited. This paper presents a smart energy management system for agricultural sensor nodes integrating a machine learning model for adaptive sampling and a batching strategy to optimize energy usage. A lightweight Stochastic Gradient Descent (SGD) regressor trained on temperature dynamics runs on-device to predict the sampling interval ($T_{s}$). In parallel, the node adjusts the number of buffered samples as the battery state of charge (SOC) decreases, reducing Long Range (LoRa) transmissions. Field experiments show that the proposed approach reduces energy consumption by 77.8% compared with fixed-interval sampling, while maintaining good temperature fidelity with Mean Absolute Error (MAE) of 0.537 °C for temperature reconstruction.

## 1. Introduction

Wireless sensor networks (WSNs) [1] have become important in modern agriculture, enabling real-time data collection for informed decision-making. Through advanced data analysis, these systems can anticipate risks, interpret trends, and provide recommendations to optimize crop production and quality [2,3,4]. In pitaya cultivation, for example, WSNs have been used to monitor soil moisture, temperature and light intensity, enabling precise irrigation, optimized harvest timing, and controlled application of artificial lighting to enhance production and sustainability and reduce operational costs [5,6,7]. However, the remote location of agricultural farms, together with infrastructure and energy limitations, means that they require a low-maintenance and energy-efficient WSN that can operate autonomously for extended periods without compromising data quality.

Conventional WSNs often rely on fixed sampling periods, which also include wireless transmissions at the same intervals. This approach leads to redundant measurements, unnecessary wireless transmissions, and excessive energy consumption, thereby reducing the lifetime of battery-powered nodes [8]. Wireless transmission is the most power-demanding operation, followed by sensor activation and local computation [9]. Minimizing sampling frequency and transmission events is critical for optimizing total energy consumption ($E_{total}$) [10]. Adaptive strategies that dynamically adjust sampling periods according to environmental variations and transmission intervals based on the node’s state of charge (SOC) are effective for achieving energy-efficient operation and extending the battery life of sensor nodes [11].

In this context, Tiny Machine Learning (TinyML) [12] provides a practical path to implement adaptive strategies directly on embedded systems such as microcontrollers (MCUs) connected to sensors and actuators [13]. By processing data locally, TinyML enables dynamic adjustment of the sampling intervals according to real-time operating conditions. This approach minimizes redundant measurements and unnecessary wireless transmissions, effectively addressing the primary sources of power consumption in WSNs, preserving relevant data while reducing both energy and memory usage. As a result, this strategy promotes the development of scalable and intelligent WSNs suitable for long-term agricultural monitoring applications.

Recent efforts to reduce the energy consumption of wireless sensor nodes have combined adaptive sampling algorithms, low-power design and TinyML for on-device intelligence. Early studies focused on dynamic sampling periods using traditional algorithms. One of the most representative examples is the Dynamic Sampling Rate Algorithm (DSRA) [14], which dynamically adjusts the sampling rate based on the change in measured variables. Despite achieving noticeable reductions in $E_{total}$, DSRA relies on fixed equations and requires manual parameter tuning. A similar work is presented in [15], where sampling frequency is modulated according to the variability of temperature and humidity. These adaptive techniques are computationally simple and effective under stable conditions; however, they cannot autonomously adapt to environmental contexts.

To overcome these limitations, several works have introduced learning-based adaptive sampling algorithms. Cecchinel et al. [16] introduced a middleware for a WSN, employing live machine learning and deep sleep cycles to extend battery life. By analyzing historical data, the system can adjust sampling and transmission periods, achieving energy savings while maintaining data quality. Subsequent developments, such as the Adaptive Sampling Edge Computing framework [17], use the most recent sensor data to perform a linear adjustment and dynamically adjust the sampling rate using an adaptive strategy based on data fluctuation. Although deployed in real prototypes, these approaches were implemented using high-end devices such as Raspberry Pi due to the complexity of the algorithms.

A more advanced research direction has explored the integration of learning techniques in WSNs to improve energy efficiency and adaptive operation. The Deep Reinforcement Learning Calibration method [18] demonstrated its potential to manage sensor resources dynamically to maximize sensor life. However, this study was limited exclusively to simulation environments. Similarly, later optimization algorithms for precision agriculture, such as Niu et al. [19], Adaptive Sensing Spatio-Temporal [20], and the HOSNA model [21], implemented machine learning models for optimizing sampling and wireless transmissions, reducing $E_{total}$ by up to 30%; however, these strategies depended on centralized processing and/or static parameterization.

Addressing the constraints of centralized processing, the adoption of on-device TinyML-based approaches represents an important development in this field. The Ambrosia system [22] presented the implementation of a lightweight algorithm running entirely on sensor nodes, achieving around 60% reduction in sampling and wireless transmissions. Similarly, tinyMAN [23] proposed an energy management framework for energy-harvesting wearable devices. A model based on reinforcement learning using TensorFlow Lite improved energy efficiency by 45%. Although this study did not apply TinyML for adaptive sampling, it successfully demonstrated the feasibility of on-device learning in low-power microcontrollers.

In summary, these studies reveal a clear progression from rule-based methods toward embedded learning models capable of autonomous energy management. However, most existing solutions still depend on offline optimization, centralized processing, or lack validation on actual battery-powered sensor nodes. To address these limitations, this work presents a smart energy management system for agricultural nodes that integrates TinyML to optimize power consumption. The system employs a lightweight, on-device Stochastic Gradient Descent (SGD) model that dynamically adjusts $T_{s}$ by analyzing current temperature trends and the time of day. Although trained offline, the model is deployed directly on the sensors and continues to update its weights during operation, allowing it to adapt to changing environmental conditions. In parallel, the data transmission batch size (α) is dynamically adjusted based on the battery’s state of charge (SOC). By coupling the sampling policy with both temperature and battery levels, the system extends node lifetime without compromising sensing accuracy. Validation on a pitaya crop over 60 days confirms the system’s suitability for agricultural environments with limited power and maintenance resources.

## 2. Materials and Methods

This section details the hardware and software designed to implement the proposed smart energy management system. It begins by outlining the system architecture, specifically the selection of low-power components and environmental sensors suitable for outdoor deployments. The methodology further describes the firmware development and the labeling strategy used to create the training dataset. Subsequently, the specific TinyML pipeline for on-device deployment of the SGD model is defined. Finally, the section establishes the experimental setup used to validate the system’s energy efficiency and reconstruction accuracy under real-world field conditions.

### 2.1. Hardware Components

**Figure 1:**
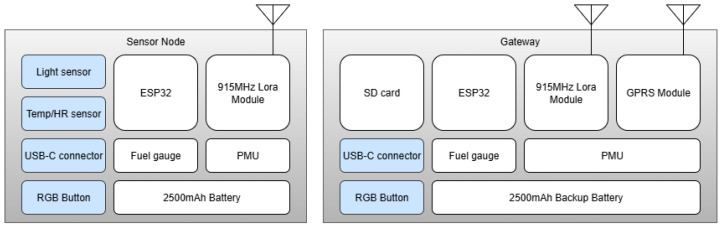
Block diagram of the proposed wireless sensor network architecture. The blocks in blue represent external peripherals. The block sensor node (**left**) integrates environmental sensors and a low-power LoRa transceiver, while the gateway (**right**) aggregates data via LoRa and forwards it to the cloud through General Packet Radio Service (GPRS) connection. Both nodes include ESP32 microcontrollers, battery level sensor (fuel gauge) and independent power management units (PMUs) for voltage regulation and battery charging.

The proposed system is based on two main components: a hardware block and a software block. The hardware block (Figure 1) consists of two sensor nodes co-located in the crop and a gateway responsible for receiving the data and forwarding them to the cloud. The software block consists of the firmware for the sensor nodes and gateway, and it also includes the online platform for data storage and presentation.

The hardware components are shown in Table 1. Each node in the network is built around an ESP32-WROOM-32U microcontroller (Espressif Systems, Shanghai, China), selected for its low power consumption in deep sleep mode and its compatibility with TinyML models. The sensor node integrates an SHT40 sensor (Sensirion AG, Stäfa, Switzerland) for reading temperature (*T*) and relative humidity (RH), and a BH1750 sensor (ROHM Co., Ltd., Kyoto, Japan) for measuring light intensity (Lx). As shown in Figure 1, these sensors are exposed to the external environment, which is why both sensors are IP65-rated, making them suitable for continuous operation in outdoor agricultural environments. Power is supplied by a 3.7 V, 2500 mAh Li-Po battery (Zhongshan Pnas Energy Technology Co., Ltd., Zhongshan, China), and the device can read the state of charge (SOC) and battery voltage using the MAX17048 fuel gauge IC (Analog Devices, Wilmington, MA, USA). For wireless communication, this WSN implemented a Reyax RYLR998 LoRa module (REYAX Technology Co., Ltd., Shenzhen, China). The gateway uses an SIM800 module (SIMCom Wireless Solutions Co., Ltd., Shanghai, China) to forward the data to the Internet via cellular uplink. All components are enclosed within a weather-resistant IP65-rated plastic housing, ensuring protection against dust and water exposure in the field.

### 2.2. Software Environment and Firmware Development

The firmware for the sensor nodes was developed using Visual Studio Code v1.111 in combination with the Arduino Community Edition Framework v0.7.2, mainly chosen for its rapid prototyping capabilities and robust support for different sensors and modules. The sensor node requires timestamp synchronization to use the hour feature for its implemented TinyML model. For this, the gateway acts as a time server, sending acknowledgments with the current timestamp to the sensor nodes after each data transmission. Timestamps are transmitted in local time (UTC-05) as UNIX epoch seconds. Upon reception, the sensor nodes update their internal RTC with the received timestamp, which can remain running even in deep sleep mode. If no acknowledgment is received for more than N consecutive transmissions (N = 3 in our deployment), the node continues using its last RTC value.

For cloud forwarding, we used Hypertext Transfer Protocol (HTTP) POST/GET on the gateway (GPRS uplink) for compatibility with the ThingsBoard Cloud Platform v4.3; because the gateway is powered by an external solar panel in our deployment, we did not optimize it for saving energy. If required, MQTT or compact binary protocols can be used to reduce power consumption.

In summary, the sensor node firmware consists of the following stages:

(a) System boot and setup.
(b) Sensor data sampling.
(c) LoRa Module initialization.
(d) Wireless Data Transmission and acknowledgment reception.
(e) Sleep mode initialization.
(f) System Sleep Mode.

On the other hand, the gateway firmware is sectioned as follows: wireless data acquisition, acknowledgment (with timestamp) transmission, and data streaming to the cloud. Two work profiles were identified based on the device activity. These profiles correspond to two different operational scenarios described as follows:

- Profile 1: Sensor sampling followed by wireless transmission. This profile consisted of the stages a–f.
- Profile 2: Sensor sampling without wireless transmission. This profile consisted of the stages a, b and f.

### 2.3. Heuristic Labeling Strategy

During a 15-day field deployment, a sensor node collected 1941 samples at 15 min intervals. Multiple environmental parameters were recorded; however, the initial training phase uses temperature (temp) and its corresponding timestamp (tms) as input features for the proposed TinyML adaptive sampling mechanism. Temperature was selected as a representative continuous signal with strong intra-day dynamics, enabling evaluation of the embedded predictive sampling framework without introducing additional multi-feature design choices.

Since the dataset lacked labels for the sampling interval, a heuristic labeling strategy was developed to generate them. The data was sorted based on the timestamp containing date and hour. In order to reduce noise (from sensor error), an exponential moving average (EMA) was applied to the temperature series. For each sample, a forward search identifies the next timestamp at which the temperature changes by at least ±0.5 °C, and the elapsed time is used as the label $T_{s}$. In this study, we set the threshold (ϵ) to ±0.5 °C because the SHT40 sensor provides typical temperature accuracy on the order of ±0.2 °C; selecting ϵ above this value reduces the likelihood that label generation is driven by sensor noise rather than physical changes. Thus, ϵ represents a practical “significant change” threshold for this deployment and sensor class.

**Figure 2:**
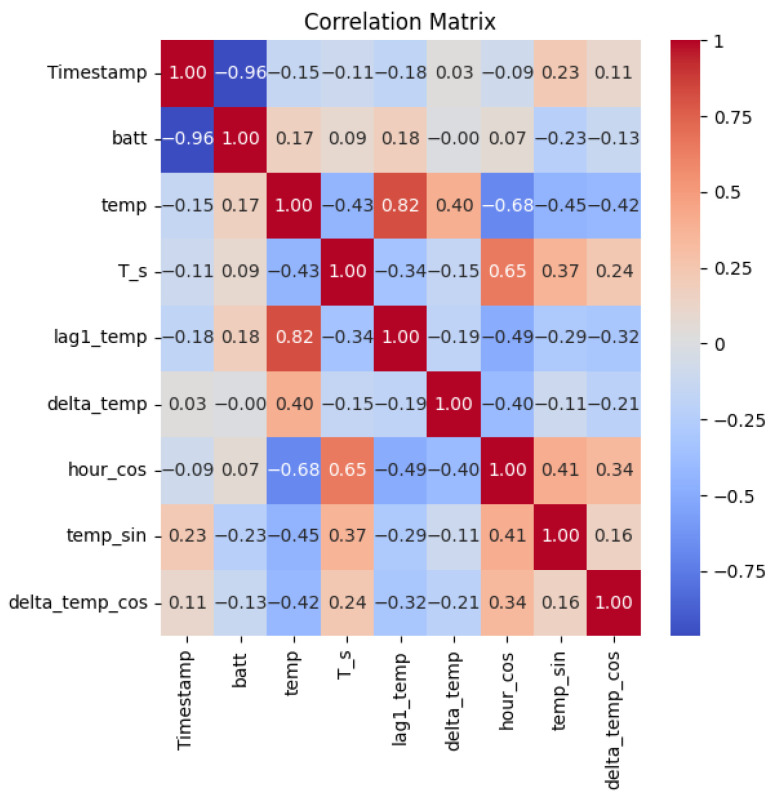
Correlation matrix for feature selection. The heatmap displays Pearson correlation coefficients between engineered features and the target sampling period ($T_{s}$). Notably, hour_cos, temp, and lag1_temp show the strongest correlations with $T_{s}$, indicating their predictive potential. Features with weak correlations were excluded from the model to enhance performance and reduce complexity.

Since target $T_{s}$ was defined, it was necessary to determine which other features could be used for the predictive process. For this, we used the Pearson linear correlation coefficient. Initially, the Pearson correlation between $T_{s}$ and *tms* was −0.111, while the correlation with *temp* was −0.43. These values indicate a weak predictive relationship, suggesting the need for feature engineering [24] to generate new variables with stronger associations to $T_{s}$. To address this, several transformations were applied: trigonometric encoding of the hour of the day (*hour_cos*) to capture periodicity; sine and cosine transformations of temperature (*temp_sin*) and its variation (*delta_temp_cos*) to model potential nonlinear patterns; and lag-based features to represent short-term historical changes. The resulting dataset consisted of the following features: *Timestamp*, *temp*, *temp_ema*, *t_samp*, *lag1_temp*, *delta_temp*, *hour_cos*, *temp_sin*, and *delta_temp_cos*. As shown in Figure 2, the correlation matrix revealed that *hour_cos* and features like temp and lag1_temp exhibited the strongest associations with $T_{s}$.

### 2.4. Machine Learning (ML) Model Design and Training

Since the goal is to predict the optimal sampling period ($T_{s}$), which is a continuous variable rather than a discrete category, a regression approach is most suitable. After evaluating various regression algorithms, the SGD regressor was selected for its computational efficiency and suitability for deployment on memory-constrained devices like ESP32. Its iterative weight update mechanism allows for incremental learning, enabling the model to adapt to new data during field operation on-device independently from the gateway.

Using Google Colab Environment v2025-11-13, the dataset was split into training and testing subsets following a 70/30 ratio (X_test, y_test, X_train, y_train), while preserving temporal order to ensure the data was continuous in time. The predictive feature set included temp, hour_cos, and lag1_temp. A pipeline was defined using scikit-learn, consisting of four main stages: (i) mean imputation in case of missing values, (ii) second-degree polynomial feature expansion to capture nonlinearities, (iii) standardization for scale normalization, and (iv) an SGD regressor with the following hyperparameters:

- Loss function: Huber. This loss function is robust to outliers, combining the advantages of both Mean Squared Error (MSE) and Mean Absolute Error (MAE).
- Regularization: Elastic Net (L1 ratio = 0.15). This regularization technique combines L1 and L2 penalties.
- Learning rate: Adaptive. This strategy adjusts the learning rate based on the model’s performance during training, allowing for faster convergence.
- Initial learning rate: 0.05.
- Maximum iterations: 100,000.
- Tolerance: 1 × $10^{-5}$.

**Figure 3:**
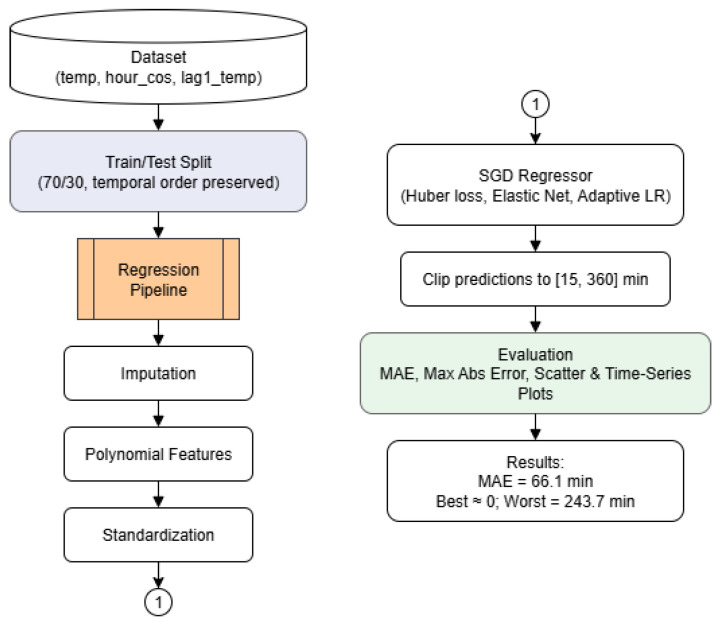
Flowchart of the TinyML model training and evaluation process. The dataset (temperature, hour cosine, and lagged temperature) is split into training and testing subsets while preserving temporal order. The regression pipeline includes imputation, polynomial feature generation, and standardization. An SGD regressor with Huber loss, Elastic Net regularization, and adaptive learning rate is used to predict the sampling period, constrained between 15 and 360 min. Model performance is evaluated using MAE, maximum absolute error, and visual time-series comparisons.

A summary of this workflow is presented in Figure 3.

The regression model predicts the optimal sampling period ($T_{s}$) as a function of current temperature and its short-term trend, as shown in Equation (1):

(1) $$T_{s}=f\left(temp,hour\_cos,temp_{t-1}\right)$$

where temp is the current temperature reading, hour_cos is the cosine transformation of the current hour, and $temp_{t-1}$ is the temperature reading from the previous sampling instant.

**Figure 4:**
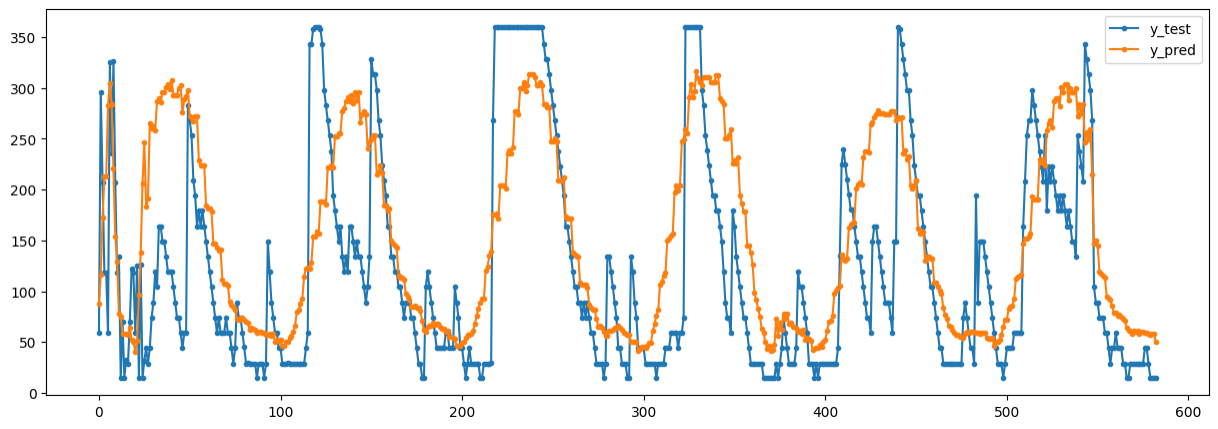
Comparison between real and predicted values of $T_{s}$. The blue line (y_test) represents the desired $T_{s}$ data, while the orange line (y_pred) corresponds to the model’s predicted output. The close alignment between both curves indicates the model’s ability to accurately capture the temporal variations of the target variable.

Model performance was evaluated using mean absolute error (MAE), including minimum and maximum absolute error. Applying the trained regression pipeline to the test set, the MAE resulted in 66.06 min, with best-case approaching zero error and worst-case reaching 243.69 min. Comparisons between predicted and true values are shown in Figure 4.

### 2.5. Sample Batch Size Adjustment Based on Battery SOC

To analyze the effect of payload size on power consumption during wireless transmission, several measurements were performed for different payload lengths ranging from 0 to 240 bytes, according to the maximum allowed by the LoRa module. The results showed a linear increase in energy consumption with payload size. However, even when no payload bytes were transmitted, the module consumed a constant 247.25 mJ, which corresponds to the baseline energy required for transmission setup and radio activation of the module. Table 2 summarizes the measured energy consumption for various payload sizes.

Considering that one complete sensor sample (temperature, humidity, light intensity, battery voltage, SOC and $T_{s}$) requires around 20 bytes, transmitting 10 samples in a single batch consumes approximately 2.85 times less energy than sending each sample individually. Therefore, adjusting the number of samples per transmission batch as the battery SOC decreases allows the system to optimize power consumption. To achieve this, the number of samples per transmission batch (α) was dynamically adjusted based on the battery SOC using the following linear relationship:

(2) $$\alpha =1+\left\lfloor \frac{\left(100-SOC\right)}{10}\right\rfloor$$

In this model, $SOC_{\min}$ and $SOC_{\max}$ represent the lower and upper battery SOC thresholds, set to 0% and 100%, respectively. Consequently, the limits for the batch size, $\alpha_{\min}$ and $\alpha_{\max}$, were set from 1 to 10 samples.

This strategy implements an adaptive transmission batching scheme: when energy reserves are low, the sensor node conserves power by reducing radio transmissions and aggregating measurements into larger batches. Specifically, Equation (2) defines α as the number of samples buffered before an LoRa transmission. This mechanism does not modify $T_{s}$; all samples are still acquired according to the TinyML-predicted $T_{s}$ and stored in a non-volatile memory. This strategy introduces a trade-off where data latency increases on the monitoring platform as battery SOC decreases; however, sensing accuracy is not compromised, as the complete historical dataset remains intact.

The transmission period can be expressed as the time required by the sensor node to collect α samples until wireless transmission is triggered. Therefore, the transmission period $T_{\mathrm{tx}}$ can be defined as:

(3) $$T_{tx}=\sum_{i=1}^{\alpha }T_{s}\left(temp_{i},hour\_cos_{i},lag1\_temp_{i}\right)$$

where $T_{s}$ represents the adaptive sampling period function determined by temperature dynamics and other features mentioned before.

### 2.6. ML Model Deployment into Microcontroller

To enable on-device inference, the trained regression pipeline was adapted for deployment on an ESP32 microcontroller. Since the model was originally implemented in scikit-learn, the relevant parameters from each pipeline stage were exported into C-compatible arrays of type float32. The array parameters extracted consist of three elements which correspond to the features temp, hour_cos and lag1_temp, respectively. These parameters are summarized as follows:

- SimpleImputer: Missing value statistics were extracted and stored as IMP_MEAN:
  (4) IMP_MEAN={16.55773163, 0.00198541, 17.32397652}
- StandardScaler: The features’ mean and scaling factors were exported as arrays SC_MEAN and SC_SCALE, respectively. These arrays correspond to the 10 features generated after the polynomial expansion stage, including second-order interactions.
  (5) $$\begin{array}{cc} \mathrm{SC}\_\mathrm{MEAN}= & \{1.00000000,\;16.55773163,\;0.09185410,\;17.32397652,\;280.93930054,\\ \; & \;\;0.31538394,\;289.95138550,\;0.47749826,\;1.11989403,\;303.71682739\} \end{array}$$
  (6) $$\begin{array}{cc} \mathrm{SC}\_\mathrm{SCALE}= & \{1.00000000,\;2.60399795,\;0.68488032,\;1.89648676,\;97.21928406,\\ \; & \;\;12.09021854,\;75.33477783,\;0.35575938,\;12.15891838,\;71.39929962\} \end{array}$$
- SGD Regressor: The trained weight vector *w* and bias term *B* were stored in arrays W and B.
  (7) $$\begin{array}{cc} W= & \{0.00000000,\;-133.09301758,\;158.57470703,\;-4.52247000,\;87.79292297,\\ \; & \;\;-141.61976624,\;16.58968163,\;21.56970406,\;32.55502319,\;0.16770068\} \end{array}$$
  (8) B=148.65100098

These arrays were embedded into the firmware, enabling the ESP32 to replicate the preprocessing and inference steps of the original pipeline. During runtime, sensor measurements are first normalized using the imputer and scaler statistics, and then passed through the SGD regressor to predict the sampling time $T_{s}$, as shown in Algorithm 1. The input array $x_{\mathrm{raw}}$ contains the raw feature values (temp, hour_cos, lag1_temp), and the output *y* represents the predicted $T_{s}$. The function safe_val() handles the mean imputation by using the parameters IMP_MEAN. The function poly2_expand() generates the second-order polynomial features, while standardize() applies the normalization using SC_MEAN and SC_SCALE. Finally, the linear prediction is computed using the weights W and bias B. The predicted output *y* (representing $T_{s}$) is clamped within the range of 15 to 180 min to avoid outliers. The function savePendingContextToNVS() stores the last processed feature vector and predicted value in non-volatile storage (NVS) for later use during model updates.
**Algorithm 1** Model Prediction Routine**Require:** Raw input vector $x_{\mathrm{raw}}=\left[x_{1},x_{2},x_{3}\right]$ **Ensure:** Predicted output *y*  1:**Mean Imputation:**  2:$x_{\mathrm{imp}}\left[i\right]\leftarrow \mathrm{safe}\_\mathrm{val}\left(x_{\mathrm{raw}}\left[i\right]\right)$  3:**Polynomial Feature Expansion:**  4:$x_{\exp}\leftarrow \mathrm{poly2}\_\mathrm{expand}\left(x_{\mathrm{imp}}\right)$  5:**Standardization:**  6:$\mathrm{LAST}\_\mathrm{XZ}\leftarrow \mathrm{standardize}(x_{\exp})$  7:**Linear Prediction:**  8:y←B  9:**for** i=0 to 9 **do**10:      y←y+W[i]·LAST_XZ[i]11:**end for**12:**Context Saving:**13:savePendingContextToNVS(LAST_XZ,y)14:**Range Clamping:**15:y←clamp(y, 15.0, 180.0)16:**return** *y*

Additionally, the model update routine shown in Algorithm 2 recalculates the weights *W* and bias *B* using the previous sensor data saved in the array LAST_XZ and the true observed sampling period $y_{\mathrm{true}}$. This last value is computed as follows:

(9) $$y_{\mathrm{true}}=\frac{0.5\;\mathrm{{}^{\circ}C}}{\Delta T}\cdot T_{s}^{\prime }$$

where ΔT is the temperature difference between the current and previous readings and $T_{s}^{\prime }$ is the previous predicted $T_{s}$. This calculation provides the actual $T_{s}$ that the sensor should experience, which is then used to update the model. While the offline model uses adaptive learning rates for training, the on-device update routine uses a simplified weight update equation to simplify the algorithm in the microcontroller. The learning rate (LR) constant is 0.01 in this algorithm.
**Algorithm 2** Model Update Routine**Require:** True output $y_{\mathrm{true}}$ **Ensure:** Updated model parameters *W* and *B*
  1:**Parameter Constants:**  2:LR = 0.01  3:L1_RATIO  4:**Parameters Retrieval:**  5:W,B← loadModelToNVS(W,B)  6:xz← getFromNVS(LAST_XZ)  7:$y_{\mathrm{pred}}\leftarrow$ getFromNVS(y)  8:**Gradient Calculation:**  9:$r\leftarrow y_{\mathrm{pred}}-y_{\mathrm{true}}$10:**if** |r|≤HUBER_EPS **then**11:       g←r12:**else**13:       g←HUBER_EPS·sgn(r)14:**end if**15:**Weights and Bias Update:**16:**for** i=0 **to** 9 **do**17:       reg←α·(L1_RATIO·sgn(W[i])+(1−L1_RATIO)·W[i])18:       $W_{updated}\left[i\right]\leftarrow W\left[i\right]-LR\cdot \left(g\cdot xz\left[i\right]+reg\right)$19:**end for**20:$B_{updated}\leftarrow B-LR\cdot g$21:**Model parameters saving:**22:saveModelToNVS($W_{updated},B_{updated}$)

### 2.7. Power Consumption Evaluation

In the proposed system, only the sensor nodes are battery-powered; the gateway is excluded from $E_{total}$ analysis because it relies on an external power source. The proposed deployment consisted of two devices: ($Sensor_{fixed}$), a sensor node with fixed $T_{s}$ of 15 min, and ($Sensor_{ML}$), a sensor node with the TinyML model implemented. Power consumption was evaluated across six operational stages (common between $Sensor_{fixed}$ and $Sensor_{ML}$) listed in Section 2.2. A TinyML model inference stage was not included in the analysis because of its low power consumption, which can be negligible compared with sensing and radio transmission.

Measurements during stages (a)–(e) were conducted using a ChargerLAB POWER-Z KM003C, a portable USB-C power analyzer functioning as both a digital voltmeter and ampmeter. Current measurements during stage (f) were conducted by using the Fluke 289 True-RMS Multimeter, which can accurately measure microampere-level currents, whereas the KM003C is limited to a milliampere resolution.

### 2.8. Data Collection

Because α defines the number of samples sent based on the battery SOC, a storage mechanism in the ESP32’s non-volatile memory (NVS) is used to safeguard readings until transmission. Under this scheme, the node continues to wake from deep sleep to capture environmental data strictly following the interval $T_{s}$ predicted by the TinyML model. However, the LoRa radio—being the most energy-intensive component—activates only once α samples have accumulated in the buffer (up to a maximum of 10). At that point, both the stored data and the current sample are sent to the gateway in a single batch transmission (payload up to 240 bytes). Therefore, the sampling resolution is preserved; the adaptation affects only the LoRa transmission schedule and the latency of server-side updates.

Each stored sample included its corresponding $T_{s}$ offset value, representing the elapsed time since the previous measurement. For the most recent sample (Profile 1), the $T_{s}$ value was set to zero. The gateway used these offsets to reconstruct the timestamp of each measurement. After receiving a payload, the gateway used its current timestamp and sequentially subtracted the $T_{s}$ values to determine the original timestamps of each sample before forwarding them to the online platform.

**Figure 5:**
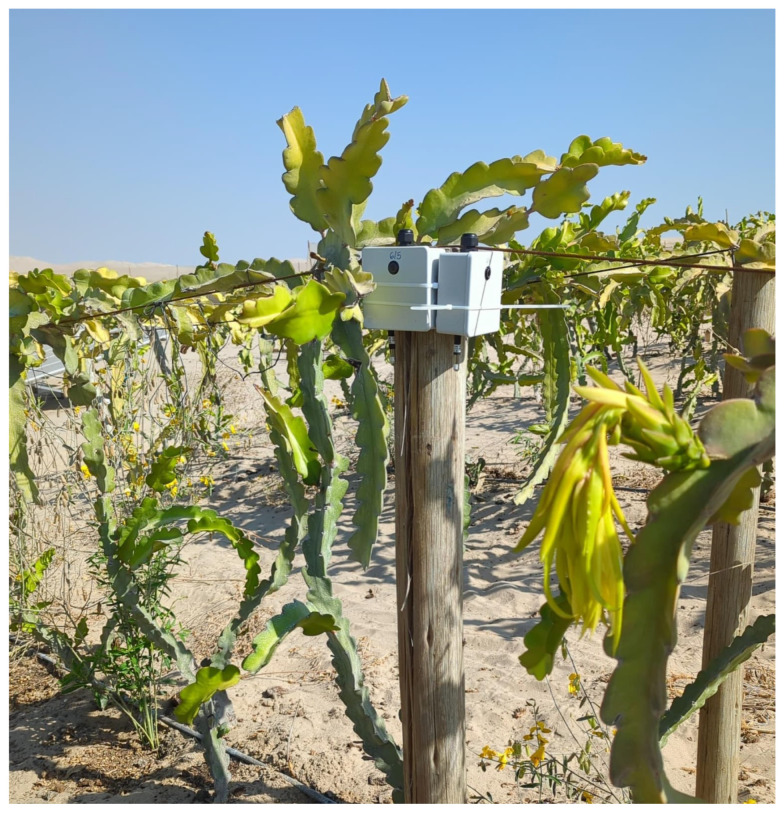
Installation setup for $Sensor_{fixed}$ (left) and $Sensor_{ML}$ (right) in a pitaya crop located in the southern coastal region of Peru.

A pilot deployment was carried out in a pitaya crop located in the southern coastal region of Peru from 3 November 2025 to 5 January 2026. Two sensor nodes were co-located in the field and operated continuously for 60 days. The recorded parameters included temperature, battery SOC and timestamp, which were used to evaluate and compare the power consumption and data loss rate between $Sensor_{fixed}$ and $Sensor_{ML}$. This setup is shown in Figure 5.

## 3. Results

This section presents the experimental results of the proposed approach, which are defined based on two sensor nodes: $Sensor_{fixed}$, which is the sensor node with a fixed $T_{s}$ of 15 min, and $Sensor_{ML}$, which is the sensor node with the proposed TinyML adaptive $T_{s}$ model. The performance was evaluated based on two metrics: $E_{total}$ and relevant data loss.

### 3.1. Energy Consumption

**Figure 6:**
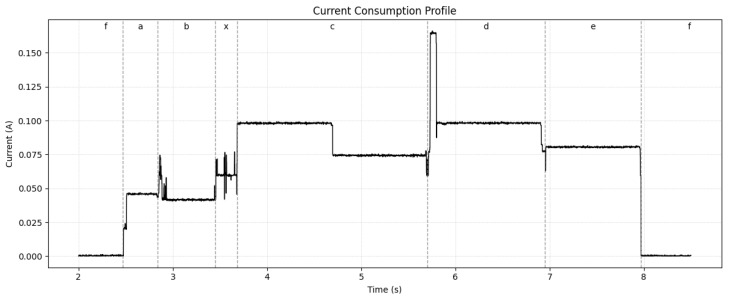
Current consumption profile across different operating states of the sensor node. The stages are represented with letters a–f mentioned previously. Stage x represents the stage where the ML model is running into the sensor. Vertical dashed lines are used to indicate the transitions between the different operating stages, which are labeled at the top of the graph.

The energy consumption of the sensor node was analyzed by measuring the current draw across its main operating states (a)–(f) presented in Section 2.2. Figure 6 shows the current profile measured by using the POWER-z meter.

Since the current presented a dynamic behavior, it is appropriate to show the total energy used during each stage. Therefore, we define $E_{i}$ as the energy consumed during each stage *i* of the sensor node operation, including stages (a)–(f) presented in Section 2.2. Based on these parameters, the total energy consumption $E_{\mathrm{profile}}$ for each operation mode can be expressed as follows:

(10) $$E_{\mathrm{Profile}\;1}=E_{a}+E_{b}+E_{c}+E_{d}+E_{e}+E_{f}$$

(11) $$E_{\mathrm{Profile}\;2}=E_{a}+E_{b}+E_{f}$$

where Profile 1 corresponds to sampling and transmission, while Profile 2 corresponds to sampling only, in which each energy component can be calculated as:

(12) $$E_{i}=\sum_{t=t_{i}}^{t_{f}}V_{t}\cdot I_{t}\cdot c$$

where *c* is the measurement period of the POWER-Z device, $V_{t}$ is the measured voltage at time *t*, $I_{t}$ is the measured current at time *t*, $t_{i}$ is the start time of each stage, and $t_{f}$ is the end time of each stage. However, since the voltage *V* and *c* remained relatively constant during each stage, Equation (12) can be simplified to:

(13) $$E_{i}=V\cdot c\cdot \sum_{t=t_{i}}^{t_{f}}I_{t}$$

where *V* is approximately 3.70 V and *c* is 0.001 s.

Despite the energy consumption during stages (a)–(e) remaining approximately constant throughout the sensor’s operation, the energy during stage *f* (sleep mode) can vary significantly based on the sampling period $T_{s}$. Therefore, for stage *f*, we can approximate its power consumption, since $I_{f}$ was considered as constant during this stage. Therefore, $E_{f}$ can be expressed as:

(14) $$E_{f}=V\cdot I_{f}\cdot T_{s}$$

where $T_{s}$ is the sampling period predicted by the TinyML model and $I_{f}$ is the current consumption during sleep mode, which is measured as 90 μA. Based on this equation, the $E_{total}$ can be calculated, since we get the energy during each stage individually. For reference, these results are shown in Table 3.

Using the measured energy values presented in Table 3, we can estimate $E_{total}$ for each work profile of the sensor node. For Profile 1 (sampling + transmission), $E_{total}$ can be expressed as:

(15) $$E_{\mathrm{Profile}\;1}=1794+0.333\cdot T_{s}\;\left(\mathrm{mJ}\right)$$

For Profile 2 (sampling only), $E_{total}$ can be expressed as:

(16) $$E_{\mathrm{Profile}\;2}=207+0.333\cdot T_{s}\;\left(\mathrm{mJ}\right)$$

Based on the data mentioned in Section 2.8, the time between measurements was calculated, which corresponds to the sampling period $T_{s}$ which was used in Equations (15) and (16). Using this information, the energy consumption $E_{total}$ of each device during the evaluation period was estimated as follows:

- **$Sensor_{fixed}$**: Since $Sensor_{fixed}$ operates with a fixed sampling period of 15 min, we first estimate a theoretical value by setting $T_{s}=900$ s in Equation (15). Therefore, the energy consumption for $Sensor_{fixed}$ can be calculated as:
  (17) $$E_{avg}^{A}=\sum_{j=1}^{N}E_{\mathrm{Profile}\;1,\;j}$$
  where *N* is the total number of samples collected during the evaluation period. By substituting N=6471 samples and calculating the $T_{s}$ as follows:
  (18) $$T_{s}=tms_{k}-tms_{k-1}\;\mathrm{for}\;k=2,3,\ldots ,N,$$
  substituting these values into Equation (17), we get:
  (19) $$E_{avg}^{A}=13,548.33\;\left(J\right)$$
  which represents 40.68% of the total battery capacity (2500 mAh at 3.7 V = 33,300 J). Therefore, based on the dataset, $Sensor_{fixed}$ started at 88.1% of battery SOC and ended at 34.7%, which represents 53.4% of the battery capacity.
- **$Sensor_{ML}$**: For $Sensor_{ML}$, the sampling periods varied according to the TinyML model predictions. It can execute Profile 1 and Profile 2 depending on the value of α. Based on this, the energy consumption for $Sensor_{ML}$ can be calculated as:
  (20) $$E_{avg}^{B}=\sum_{i=1}^{M}E_{i}$$
  where *M* is the total number of samples during the evaluation period and $E_{i}$ is defined as:
  (21) $$E_{i}=\left\{\begin{array}{cc} E_{\mathrm{Profile}\;1}, & \mathrm{if}\;i\;\mathrm{mod}\;\alpha =0\;\\ E_{\mathrm{Profile}\;2}, & \mathrm{otherwise}\; \end{array}\right.$$
  where *i* is the sample index (from 1 to *M*) and α is the number of samples per transmission batch, which depends on the battery SOC. Analyzing the dataset, the battery SOC ranged from 89.6% to 80.6%. Therefore, the value of α was two samples per transmission. Therefore, for every two samples collected, the device executed Profile 1 (sampling + transmission) once and Profile 2 (sampling only) once.
  By substituting M=2050 and calculating the $T_{s}$ for each sample from the dataset as in Equation (18), substituting these values into Equation (20), the following was obtained:
  (22) $$E_{avg}^{B}=3002.60\;\left(J\right)$$
  representing 9.0% of the total battery capacity. Therefore, the real battery capacity percentage, based on the dataset, shows that the $Sensor_{ML}$ started at 89.6% of battery SOC and ended at 80.6%, which represents a 9.0% of the battery capacity.

**Figure 7:**
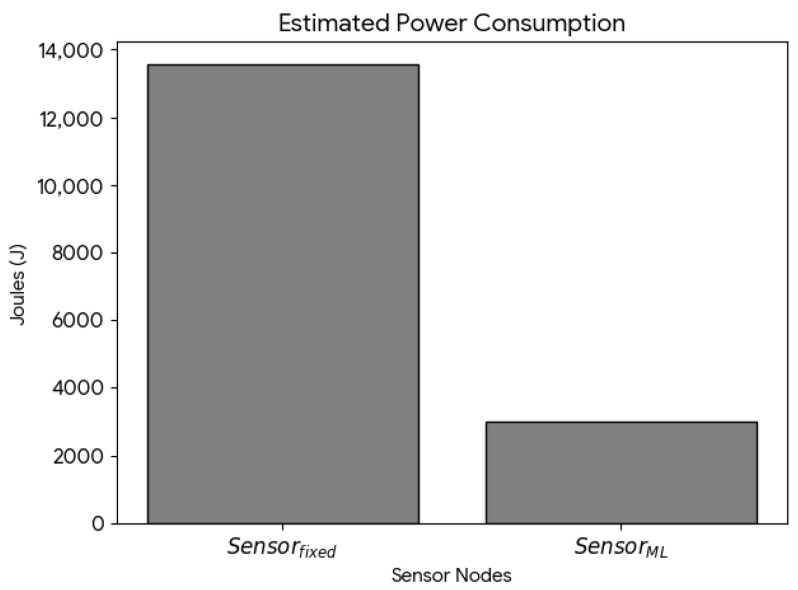
Estimated power consumption profiles of the fixed 15 min sampling node and the TinyML adaptive node. The TinyML-based adaptive sampling approach demonstrates a significant reduction in energy consumption compared to the fixed sampling strategy.

Figure 7 illustrates the energy consumption reduction for $Sensor_{fixed}$ and $Sensor_{ML}$. The results confirm that the proposed system with TinyML-based adaptive sampling ($Sensor_{ML}$) achieved a significant reduction in energy consumption compared to the fixed-interval sampling approach ($Sensor_{fixed}$). Specifically, $Sensor_{ML}$ consumed approximately 77.8% less energy over the evaluation period. This reduction is primarily attributed to the adaptive sampling strategy combined with the implementation of the α parameter.

Beyond the fixed-interval baseline, we contrast this proposal against representative approaches from the literature. Table 4 compares these approaches with this work in terms of achieved energy reduction. Compared to rule-based adaptive methods [15] and centralized methods [21], this work achieves comparable and higher energy savings while removing dependence on a central node for the TinyML model. Relative to Ambrosia [22] and tinyMAN [23], the results of this paper are in the same range of energy reduction. It is important to mention that the evaluation was conducted on battery-powered nodes under real field conditions.

### 3.2. Reconstruction Accuracy

To evaluate the effectiveness of the TinyML-based adaptive sampling strategy in preserving temperature trends, it was first necessary to calculate the *Sampling Reduction Ratio* (SRR), which quantifies the reduction in the number of samples collected by $Sensor_{ML}$ relative to $Sensor_{fixed}$. It is defined as:

(23) $$\mathrm{SRR}=1-\frac{N_{B}}{N_{A}}$$

where $N_{A}$ is the number of valid measurements from the $Sensor_{fixed}$, and $N_{B}$ corresponds to the number of measurements from node B.

During the real field deployment, the fixed node collected $N_{A}=6471$ samples, while the TinyML node acquired $N_{B}=2050$ samples. Substituting into (23), the resulting SRR was:

(24) $$\mathrm{SRR}=1-\frac{2050}{6471}\approx 0.683$$

indicating that approximately 68.3% of potential samples were omitted by the adaptive strategy, therefore reducing transmission and energy usage.

After the SRR analysis, the information preservation of the adaptive sampling approach was evaluated by using the Mean Absolute Error (MAE), which measures the average magnitude of errors between the reconstructed temperature values. This metric is defined as follows:

(25) $$\mathrm{MAE}=\frac{1}{N}\sum_{k=1}^{N}\left|temp_{A}\left(t_{k}\right)-temp_{B}\left(t_{k}\right)\right|$$

where $temp_{A}\left(t_{k}\right)$ is the temperature reading from $Sensor_{fixed}$ at timestamp $t_{k}$, $temp_{B}\left(t_{k}\right)$ is the corresponding reconstructed temperature from the adaptive $Sensor_{ML}$ at the same timestamp, and *N* is the total number of timestamps evaluated.

**Figure 8:**
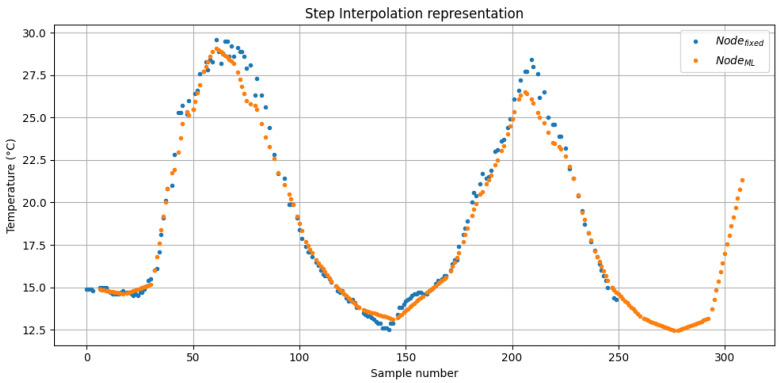
Comparison of temperature readings between $Sensor_{fixed}$ and $Sensor_{ML}$ using linear interpolation, which evaluates the reconstruction accuracy for past data. The figure shows the first 250 samples only.

**Figure 9:**
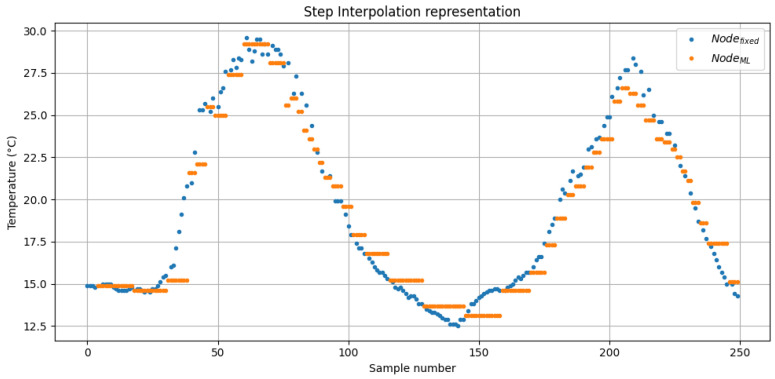
Comparison of temperature readings between $Sensor_{fixed}$ and $Sensor_{ML}$ using step interpolation, which evaluates the reconstruction accuracy during real-time monitoring. The figure shows the first 250 samples only.

The temperature readings of the adaptive $Sensor_{ML}$ (temp4) were compared to those of the reference $Sensor_{fixed}$ (temp2) using two reconstruction methods:

- Linear interpolation: This method estimated intermediate values as straight lines between measurements, representing how well the system could reconstruct the past. For each data point of $Sensor_{fixed}$, the corresponding value from $Sensor_{ML}$ was interpolated linearly as follows:
  (26) $$temp_{B}\left(t\right)=temp_{B}\left(t_{i}\right)+\frac{temp_{B}\left(t_{i+1}\right)-temp_{B}\left(t_{i}\right)}{t_{i+1}-t_{i}}\cdot \left(t-t_{i}\right)\;\mathrm{for}\;t\in \left[t_{i},t_{i+1}\right]$$
  where *t* is the timestamp of the $Sensor_{fixed}$ measurement, and $t_{i}$ and $t_{i+1}$ are the timestamps of the nearest previous and next measurements from $Sensor_{ML}$, respectively. With this method, it was possible to calculate each temperature value for $Sensor_{ML}$ at the same timestamp as the data point from $Sensor_{fixed}$. Therefore, the MAE calculated using Equations (25) and (26) resulted in 0.537 °C with a maximum error of 4.55 °C. The comparison between both sensors using this interpolation method is shown in Figure 8.
- Step interpolation: This method maintained constant values between consecutive measurements, representing the accuracy perceived by users during real-time monitoring. For each data point of $Sensor_{fixed}$, the corresponding value from $Sensor_{ML}$ was interpolated as follows:
  (27) $$temp_{B}\left(t\right)=temp_{B}\left(t_{i}\right)\;\mathrm{for}\;t\in \left[t_{i},t_{i+1}\right]$$
  where *t* is the timestamp of the $Sensor_{fixed}$ measurement and $t_{i}$ is the timestamp of the nearest previous measurement from $Sensor_{ML}$. Using this method, the MAE calculated using Equations (25) and (27) resulted in 0.811 °C with a maximum error of 13.8 °C. The comparison between both sensors using this interpolation method is shown in Figure 9.

These results demonstrate that the TinyML-based $Sensor_{ML}$ maintains a high reconstruction accuracy despite a 68.3% reduction in samples. With linear interpolation, the MAE is 0.537 °C, which is consistent with the sensor’s accuracy (±0.2 °C) plus residual noise. With step interpolation, the MAE remains 0.811 °C, meaning the value presented to users deviates on average by less than 1 °C. Therefore, the adaptive sampling strategy, combined with the SOC-driven parameter α, captures the essential temperature dynamics while reducing sampling and transmissions.

## 4. Discussion

We presented a TinyML-based smart energy management system for battery-powered agricultural sensor nodes. By integrating an on-device SGD model to dynamically adjust sampling periods based on temperature conditions, the proposed system significantly reduced power consumption while maintaining high temperature reconstruction accuracy. Furthermore, by incorporating a dynamic batching approach based on battery SOC, the system further optimized energy usage during wireless communication.

A fundamental distinction of this work lies in its energy mechanics compared with traditional rule-based strategies. Such systems typically must wake up at frequent intervals to sample sensors and check whether a threshold has been crossed; this approach consumes significant energy during the boot ($E_{a}$) and sensing ($E_{b}$) stages, even when the collected data are redundant and ultimately discarded. In contrast, our TinyML approach uses an on-device SGD model to estimate the sampling period ($T_{s}$). Rather than waking up to check for change, the model predicts the duration until a significant temperature variation (defined as ±0.5 °C) is expected. This allows the sensor node to remain in deep sleep ($E_{f}$) for extended durations without unnecessary wake-up cycles. This shift from “reactive checking” to “on-device prediction” is the primary driver of the reduction in total energy consumption. As future work, we will include a systematic comparison against reactive rule-based strategies (e.g., threshold/derivative checking), either through controlled experiments under identical deployment conditions or via simulation that replays measured temperature dynamics.

Our experimental results demonstrate that the proposed system achieves a 68.3% reduction in sampling compared to a fixed 15 min interval, while maintaining an MAE of only 0.537 °C for linear temperature reconstruction. These findings confirm that the adaptive approach significantly extends sensor node lifetime without compromising data fidelity. Although the $T_{s}$ prediction MAE (66.06 min) is high relative to the 15–360 min operational window, this metric evaluates interval timing accuracy rather than the reconstruction fidelity of the sensed signal. In our system, the primary objective is to preserve monitoring fidelity through accurate temperature reconstruction, which remains low on average (reconstruction MAE of 0.537 °C using linear interpolation). However, the large $T_{s}$ MAE reveals an important limitation: the model occasionally overestimates stable periods, delaying the next sampling event and thereby increasing interpolation error. This behavior is consistent with the observed reconstruction outliers (maximum errors of 4.55 °C for linear and 13.8 °C for step interpolation), even though such events are infrequent.

In terms of scalability, the SGD regressor architecture described in Section 2.4 is not limited to temperature-only operation. The same learning pipeline can be retrained using alternative or multivariate inputs (e.g., RH, illuminance, or [temp, RH, lux]) to predict $T_{s}$. Although incorporating additional features may improve context awareness, it also increases model and preprocessing requirements, such as feature engineering and additional hyperparameter tuning. Therefore, this study focuses on single-feature validation to establish the feasibility of the TinyML adaptive sampling methodology, leaving systematic multivariable evaluation as future work. We also note that selecting ϵ based on agronomic criteria and performing a sensitivity analysis are important next steps. Future work will, therefore, evaluate application-driven thresholds (e.g., defined by crop-management relevance) and quantify how varying ϵ affects the label distribution, predicted $T_{s}$, and the resulting energy consumption. Overall, the proposed system demonstrated a promising step toward long-term, low-maintenance, and energy-efficient monitoring for real agricultural deployments where energy resources are limited.

## Figures and Tables

**Table 1:** Summary of hardware components and system features.

Component/Feature	Description
Sensors	SHT40 (*T* and RH), BH1750 (Lx), IP65-rated
Microcontroller	ESP32-WROOM-32U 16 MB Flash memory
Power Supply	3.7 V, 2500 mAh LiPo battery
Power Management Unit (PMU)	IP2312 Li-Po charger, RT9080 3.3 V LDO
Battery Level Monitoring	MAX17048 fuel gauge IC
Wireless Communication	Reyax RYLR998 LoRa module
Internet Connectivity	SIM800 2G GPRS module
Enclosure	IP65-rated weather-resistant plastic housing

**Table 2:** Payload energy consumption for different payload sizes.

Payload (Bytes)	Energy (mJ)
0	247.25
1	251.38
20 (1 sample)	329.89
100	660.45
220 (10 samples)	1156.29
240 (max. allowed)	1238.93

**Table 3:** Measured energy consumption across the operating stages of the sensor node.

Stage	Energy (mJ)	Notes
$E_{a}$	44	Device boot and setup
$E_{b}$	163	Sensors are measured
$E_{c}$	269	LoRa module start
$E_{d}$	987	Data Transmission & Acknowledgment
$E_{e}$	331	Preparing to sleep
$E_{f}$	$0.333\cdot T_{s}$	Whole device in sleep mode

**Table 4:** Comparison with representative adaptive sampling approaches.

Approach	Energy Reduction
Temp/Humidity adaptive [15]	11.04%
Adaptive Edge Sampling [17]	12.86%
HOSNA [21]	24%
Ambrosia [22]	50%
tinyMAN [23]	45%
This work (TinyML + batching)	77.8%

## Data Availability

The data presented in this study will be made available by the authors.
